# Microplastics and probiotics: Mechanisms of interaction and their consequences for health

**DOI:** 10.3934/microbiol.2025018

**Published:** 2025-06-09

**Authors:** Jean DEMARQUOY

**Affiliations:** Université de Bourgogne Europe, Institut Agro-INRAe, UMR PAM, 21000 Dijon, France

**Keywords:** Microplastics, probiotics, oxidative stress, gut microbiota, epithelial barrier integrity

## Abstract

Microplastics (MPs), synthetic polymer particles less than 5 mm in size, are an emerging contaminant with implications for both human and ecosystem health. Being widespread in food and water sources, MPs can disrupt gastrointestinal integrity, alter the microbiota composition, and provoke oxidative and inflammatory responses. Probiotics, live microorganisms known for their gut health benefits, are now being explored for their ability to mitigate these effects. This review synthesizes evidence from *in vitro* and *in vivo* studies on how MPs impact probiotic viability, adhesion, and biofilm formation, and how certain strains may counter MP-induced toxicity by modulating oxidative stress, immune function, and the epithelial barrier integrity. Additionally, this manuscript discusses emerging applications in environmental microbiology, such as the potential use of native and engineered probiotics for microplastic bioremediation. Although the current data highlight promising avenues, key gaps remain in our understanding of strain-specific mechanisms, long-term efficacy, and real-world applicability. Addressing these will be essential to advance probiotic-based strategies in both human and environmental contexts.

## 1. Introduction

Microplastics (MPs) are synthetic polymer particles smaller than 5 millimeters. They are persistent environmental pollutants with growing relevance to human and ecological health. MPs are ubiquitous in terrestrial, freshwater, and marine ecosystems and increasingly in the human food chain [1]. Their detection in drinking water, seafood, fruits and vegetables, table salt, and even breast milk [2] highlights their widespread presence in everyday life [3],[4].

Upon ingestion, MPs interact with the gastrointestinal tract, where they may persist, translocate, or interfere with the host's biology. In animal models, MPs have been shown to disrupt gut epithelial integrity, alter the composition and diversity of the gut microbiota, induce oxidative stress, and provoke local and systemic inflammation [5].

Probiotics are defined by the Food and Agriculture Organization (FAO) and World Health Organization (WHO) as “live microorganisms which, when administered in adequate amounts, confer a health benefit on the host” [6]. Common probiotic strains include species of *Lactobacillus*, *Bifidobacterium*, *Bacillus*, *Saccharomyces*, and *Enterococcus*. They are widely recognized for their ability to support gut health, immune function, and metabolic balance [7]. Probiotics, which are traditionally used to prevent or manage gastrointestinal disorders, are also being explored for their capacity to mitigate the effects of various environmental toxicants, such as heavy metals, bisphenol A (BPA), and mycotoxins [8]. However, their role in modulating or resisting microplastic-induced toxicity is still poorly understood.

This narrative review explores the bidirectional relationship between microplastics and probiotics, thereby considering both health-related and environmental dimensions. It examines how an exposure to microplastics may influence the probiotic viability, colonization, and functionality, while also considering the potential of probiotics to counteract the oxidative stress, inflammation, and microbiota imbalances associated with microplastic exposure.

## 2. Microplastics: Exposure routes and health implications

### 2.1. Microplastics: Nature, size, and classification

Their presence in environmental and biological systems has attracted significant attention due to their ubiquity, persistence, and potential impact on ecosystems and human health [9],[10]. The small size and high surface-area-to-volume ratio make MPs especially concerning due to their potential to cross biological barriers and interact at the cellular level [11].

The chemical composition of microplastics typically includes common polymers such as polyethylene (PE), polypropylene (PP), polyvinyl chloride (PVC), polystyrene (PS), polyethylene terephthalate (PET), and polyamide (PA). These materials vary in density, crystallinity, and hydrophobicity, which influence their environmental behavior and degradation potential.

Primary microplastics include plastic pellets used in industrial manufacturing, as well as microbeads found in personal care products and cleaning agents. In contrast, secondary microplastics are generated through physical, chemical, and biological processes such as mechanical abrasion, UV-induced photodegradation, and microbial activity acting on discarded plastic waste over time [12].

The term “microplastic” encompasses a wide range of particle sizes. Broadly, microplastics range from 1 micrometer (µm) to 5 millimeters (mm) in size. Within this range, particles are often categorized as large microplastics (1–5 mm), medium microplastics (100 µm to 1 mm), and small microplastics (1–100 µm). Particles smaller than 1 µm are typically referred to as nanoplastics, though they are sometimes considered part of the microplastic continuum due to their shared origin and ecological relevance. The size of these particles directly affects their environmental transport, persistence, and bioavailability. In particular, microplastics below 10 µm are of a special concern because they are small enough to be taken up by cells and may even cross epithelial barriers, thus entering systemic circulation and internal organs [13].

It is important to note that many *in vitro* and *in vivo* studies have focused on nano-sized microplastics (less than 1 µm), which can be internalized by intestinal cells, cross epithelial barriers, and potentially reach the systemic circulation. In contrast, larger microplastics (0.1–5 mm), which dominate environmental samples, are less likely to be absorbed and may locally exert their effects by disrupting the gut barrier integrity, altering the microbial composition, or acting as carriers of toxins [14]. This size-dependent behavior must be considered when interpreting experimental results, as nano-scale particles may overrepresent bioavailability and toxicity compared to typical human exposures that involve larger, less mobile microplastics.

Additionally, microplastics exhibit a wide variety of physical and chemical characteristics. Their shapes can include irregular fragments, filaments, films, foams, or spherical beads. Over time, their surfaces become weathered, thus developing cracks, roughness, and oxidized functional groups that influence their reactivity and capacity to adsorb pollutants [15]. Furthermore, microplastics often accumulate biofilms, communities of microorganisms that colonize plastic surfaces, creating what is referred to as the “plastisphere” [16]. These biofilms can further alter the transport and degradation behavior of microplastics in aquatic and terrestrial environments.

In addition to their polymeric matrix, microplastics can contain chemical additives such as plasticizers, flame retardants, stabilizers, and pigments, which may leach into the surrounding environments [17]. Moreover, their hydrophobic surfaces can adsorb environmental contaminants such as persistent organic pollutants (POPs) and heavy metals. This dual role, as carriers of both inherent and adsorbed toxicants, raises concerns about the role of microplastics as vectors of chemical exposure to organisms, particularly in aquatic ecosystems [18].

Due to their small size and floating nature, microplastics are found across all environmental compartments, including marine and freshwater systems, soils, air, and even within the biota. Their ingestion has been documented in a wide array of organisms, from plankton and invertebrates to fish, birds [19], and mammals [20]. Potential biological effects include physical blockage, inflammation, oxidative stress, and altered energy metabolism. In humans, while research is still emerging, evidence suggests a possible accumulation in tissues, thus raising questions about chronic exposure and health outcomes [21].

The growing body of research on microplastics highlights the complexity of their environmental behavior and interactions with biological systems. A clear and consistent definition, along with standardized methodologies for sampling, analysis, and reporting, is essential to advance our understanding of their risks and informing effective mitigation strategies [22].

### 2.2. Sources and exposure pathways

**Figure 1.**
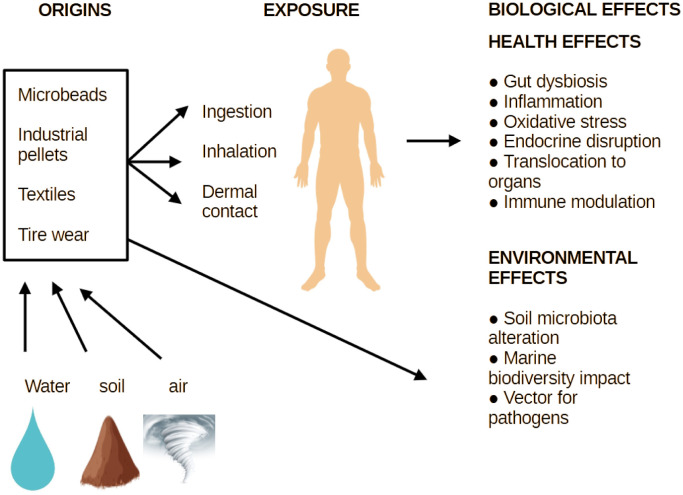
Origins, exposure routes, and biological effects of microplastics. Microplastics originate from various sources including microbeads, industrial pellets, textiles, and tire wear; they disseminate through environmental matrices such as water, soil, and air. Human exposure primarily occurs via ingestion, inhalation, and dermal contact. Once internalized, microplastics may induce a range of health effects such as gut dysbiosis, inflammation, oxidative stress, endocrine disruption, translocation to distant organs, and immune modulation. In parallel, microplastics alter the soil microbiota, disrupt the marine biodiversity, and act as vectors for environmental pathogens, thus raising concerns for both the ecosystem and human health.

Microplastics enter the human body primarily through ingestion, with lesser contributions from inhalation and dermal contact [23] (Figure 1). Microplastics have been detected in a wide range of commonly consumed food and beverage products. Both bottled and tap water are known to contain MPs, particularly polyethylene terephthalate (PET) and polypropylene (PP) fibers and fragments [24],[25]. Seafood, especially filter-feeding organisms such as mussels and oysters, can accumulate MPs in their tissues, which may then be ingested intact by humans [26]. Moreover, MPs have been identified in agricultural products including fruits, vegetables, and grains, which are likely due to irrigation with contaminated water or the use of sewage sludge as fertilizer. Additionally, MPs have been found in table salt [27], honey [28], and various dairy products [29], thus highlighting the widespread and often unavoidable nature of dietary microplastic exposure. Infants may be at an even higher risk due to plastic-containing feeding bottles and formula preparation methods that release substantial MP loads [30].

Once ingested, MPs interact with the mucosal environment of the gastrointestinal tract. Smaller MPs, especially nanoplastics, may penetrate the gut barrier, thus entering circulation and potentially accumulating in tissues. *In vivo* studies have revealed the presence of MPs in the liver, kidney, brain, and even reproductive tissues in animals [31].

### 2.3. Health effects of microplastics

Experimental data from rodent, zebrafish, and invertebrate models have demonstrated that microplastics can exert multiple detrimental effects on gastrointestinal and systemic physiology. An exposure to MPs disrupts the gut microbiota composition, often decreasing beneficial genera such as *Lactobacillus* and *Bifidobacterium* while promoting the growth of opportunistic pathogens, thereby contributing to dysbiosis [32]. Additionally, MPs can induce epithelial damage by reducing the expression of tight junction proteins (*e.g*., occludin, claudin-1), which results in an increased intestinal permeability and a compromised barrier function [33]. Immunologically, MP exposure stimulates inflammatory responses, which is marked by elevated levels of pro-inflammatory cytokines such as IL-6 and TNF-α and an increased immune cell infiltration into the intestinal tissues [34]. MPs promote oxidative stress by elevating the reactive oxygen species (ROS) levels and lipid peroxidation markers such as malondialdehyde (MDA), while impairing mitochondrial integrity [35].

Additionally, MPs may function as vectors for adsorbed contaminants, including persistent organic pollutants (POPs), heavy metals, antibiotics, and microbial pathogens. This “Trojan horse” effect enhances their toxicological complexity. An important open question is whether probiotics that bind MPs might inadvertently facilitate the delivery of sorbed toxicants (*e.g*., heavy metals, POPs) or pathogenic microbes. While the current data emphasize the protective potential of probiotics, future studies must assess whether microbe–MP complexes could shift the toxicant bio accessibility or modulate the gut uptake mechanisms. This dual-edged possibility should be considered in the development of probiotic-based interventions [36],[37].

### 2.4. Relevance to gut microbiota and probiotics

The gastrointestinal tract is the first major interface between MPs and the host's biology. The gut microbiota is sensitive to perturbation in the luminal environment. Dysbiosis induced by MPs may impair immune function, increase disease susceptibility, and compromise metabolic and neurological health [38]. Given the central role of the microbiota in maintaining homeostasis, interventions aimed at restoring microbial balance, such as probiotic supplementation, are gaining interest. Understanding how MPs interact with probiotics and whether probiotics can mitigate MP-induced gut damage represents a novel and urgent research priority.

## 3. Probiotics and gut health: Role in combating pollutants

Probiotics are live microorganisms that confer health benefits to the host. Beyond their classical health benefits, probiotics have demonstrated the capacity to mitigate toxic effects of various environmental and dietary contaminants. Several *Lactobacillus* and *Bifidobacterium* strains have been shown to bind and detoxify heavy metals such as lead, cadmium, and mercury, primarily via their cell surface components (*e.g*., teichoic acids, extracellular polysaccharides). Additionally, probiotics can sequester or degrade xenobiotics such as bisphenol A (BPA), phthalates, and certain pesticides [39],[40].

Microplastics represent a class of contaminants whose interactions with probiotics are only beginning to be understood. Given their capacity to adsorb chemical pollutants and induce oxidative and inflammatory stress in the host, MPs present multiple challenges to probiotic survival and function [41]. Conversely, based on their known interactions with other xenobiotics, probiotics may serve as bioactive shields against MP toxicity. Whether they act via direct binding to MPs, the modulation of gut barrier function, antioxidant activity, or the restoration of dysbiotic microbial communities, probiotics could emerge as key players in mitigating microplastic-related health risks. Understanding these interactions is essential to develop functional interventions for populations exposed to MPs.

## 4. Microplastics' impact on probiotic viability and function (*in vitro* studies)

### 4.1. Exposure of probiotic cultures to microplastics

*In vitro* studies have provided critical insights into how microplastics may directly affect probiotic organisms. These experiments typically expose pure cultures of probiotics to polystyrene (PS), polyethylene (PE), or polypropylene (PP) microparticles or nanoparticles under controlled conditions, thereby evaluating parameters such as bacterial growth, viability, oxidative stress markers, and adhesion properties. Teng *et al*. (2024) screened 784 bacterial strains for their ability to adsorb 0.1 µm polystyrene particles, and identified strains such as *Lacticaseibacillus paracasei* DT66 and *Lactiplantibacillus plantarum* DT88 with significant adsorption capabilities *in vitro* [42].

Multiple studies have reported that an exposure to MPs, particularly polystyrene microparticles in the range of 1–50 µm, can lead to dose-dependent reductions in the growth rate and survival of probiotic strains, including *Lactobacillus plantarum*, *Lactobacillus rhamnosus*, and *Bacillus subtilis* [43],[44]. These adverse effects are likely mediated through physical interactions between MPs and bacterial membranes, a disruption of metabolic processes, or oxidative stress due to the generation of ROS. The study of Athulya *et al*. (2024) investigated the impact of polystyrene MPs (PS-MPs) on *Bacillus tropicus* ACS1, a probiotic bacterium isolated from the gut of *Oreochromis mossambicus*. The findings revealed that an exposure to varying concentrations of PS-MPs significantly altered the bacterial growth kinetics and increased the cell viability. Additionally, there was an upregulation in the production of exopolysaccharides (EPS) and the induction of oxidative stress markers, including elevated levels of ROS, superoxide dismutase (SOD), and catalase enzymes [45].

Similarly, Shi *et al*. (2025) demonstrated that *Lactobacillus plantarum* strains with varying antioxidant capacities and affinities for PS-NPs could mitigate the toxicity induced by PS-MPs. The study highlighted that the antioxidant capabilities of *Lactobacillus plantarum* reduced oxidative damage caused by a PS-MPs exposure, and their binding affinity for PS-NPs decreased the body's PS-MPs content by increasing fecal excretion. Notably, even strains with a low antioxidant activity and binding affinity were able to reduce the toxicity, potentially through repairing the intestinal barrier and modulating the bile acid metabolism [44].

### 4.2. Biofilm formation and adhesion changes

Biofilm formation is a critical survival strategy for many probiotics, enabling them to effectively colonize the gut epithelium. An exposure to MPs can influence this process by stimulating the production of extracellular polymeric substances (EPS) and promoting surface adhesion. *In vitro* experiments have demonstrated that *Lactiplantibacillus plantarum* and *Bacillus subtilis* increase biofilm formation when exposed to MPs, likely due to surface-associated stress cues [46].

Conversely, other studies suggested that MPs can interfere with the adhesion of probiotics to intestinal epithelial cells. This may occur through the physical occupation of adhesion sites, an alteration of bacterial surface proteins, or the sequestration of adhesion-promoting nutrients. Experiments using Caco-2 or HT-29 intestinal epithelial cell lines co-cultured with MPs and probiotics have reported reduced adhesion of *Lactobacillus rhamnosus* GG in the presence of MPs [47].

Such observations raise concerns that MPs in the gastrointestinal tract may hamper the colonization efficiency and persistence of probiotics, thus potentially diminishing their therapeutic efficacy. A systematic comparison of *Bifidobacterium* versus *Lactobacillus* strains (particularly regarding their differential abilities to bind MPs, modulate inflammation, or restore barrier function) would significantly strengthen its translational value. Furthermore, the discussion overlooks the potential adverse effects of certain strains under MP exposure, such as the oxidative stress exacerbation observed in some *Bacillus* species [45]. This gap is particularly important given that MP-induced gut dysbiosis may create an environment where typically beneficial strains could exhibit unpredictable behaviors. Including such comparative analyses and safety considerations would provide a more balanced perspective for both research and clinical applications.

### 4.3. Limitations and relevance of in vitro models

While *in vitro* studies have provided valuable mechanistic insights, they often rely on experimental conditions that do not accurately reflect real-world exposure scenarios. Most studies use pristine, monodisperse MPs at relatively high concentrations, which substantially differ from the chronic, low-dose exposure experienced by humans through the daily ingestion of environmentally weathered and chemically complex MP mixtures. These real-world MPs are often aged, fragmented, biofilm-coated, and contain adsorbed contaminants that influence their biological behavior. These particles lack eco-coronas, an altered surface chemistry, or microbial colonization typical of environmental MPs [48]. Consequently, the interaction patterns observed under *in vitro* conditions may not accurately mirror those in food matrices or living organisms. Moreover, pure-culture systems exclude critical host factors such as immune responses, peristalsis, mucus dynamics, and microbiota–microbiota interactions, all of which influence the probiotic viability and function. Therefore, an interpretation of the results must be cautious and always contextualized with respect to these limitations.

Consequently, findings from simplified laboratory models must be interpreted with caution. Future studies should aim to replicate environmental exposure more faithfully by incorporating aged MPs, diverse particle types and sizes, and realistic exposure levels, while accounting for dietary and microbiome variability.

Furthermore, *in vitro* conditions lack the host immune response, mucus layer, and interactions with other microbial species that would modulate the probiotic function and survival *in vivo*. As such, while the evidence suggests that MPs can impair the probiotic viability and adhesion, these findings must be cautiously interpreted and validated in more physiologically relevant models.

## 5. *In vivo* interactions: Gut microbiome changes under microplastic exposure

### 5.1. Microplastic-induced gut dysbiosis

Animal studies, particularly in rodents, have consistently shown that MP exposure alters the gut microbiome and induces intestinal dysfunction [49]. An oral administration of MPs (typically polystyrene or polyethylene, in the size range of 1–20 µm) leads to significant changes in microbial composition, which is commonly characterized by reductions in beneficial commensals and increases in opportunistic or pro-inflammatory species [50],[51]. In this publication (and the erratum), the authors investigated the effects of an oral exposure to polypropylene MPs (PP-MPs) in mice, and revealed that ingestion causes colonic apoptosis and intestinal barrier damage through oxidative stress and inflammation [50],[51]. Their findings demonstrate that PP-MPs disrupt gut health by triggering cellular damage and impairing barrier function, thus highlighting significant toxicological risks associated with MP contamination.

Additionally, the level of systemic inflammatory markers (IL-6, TNF-α) are elevated in MP-exposed animals, along with oxidative stress markers such as malondialdehyde (MDA). This suggests that MPs may exert both local and systemic immunotoxic effects via gut-mediated pathways [52]. While rodent studies are essential to identify the potential biological effects, differences in the gut anatomy, immune responses, and microbiota composition limit the direct extrapolation to human physiology. Furthermore, most studies use acute or high-dose MP exposures that may not reflect chronic, low-level ingestion which is typical in human populations.

### 5.2. Probiotic modulation of microplastic toxicity

Emerging studies revealed that probiotic supplementation can counteract many of the adverse effects of MPs *in vivo*. In mouse models, a co-administration of probiotics has been shown to mitigate MP-induced gut inflammation, restore microbiota diversity, and preserve the intestinal barrier function. *In vivo* experiments demonstrated that mice treated with these probiotics exhibited a 34% increase in PS excretion rates and a 67% reduction in residual PS particles within the intestine [42]. The administration of *Lactiplantibacillus plantarum* DT88 mitigated PS-induced intestinal inflammation [42]. A study explored the effects of PS-MPs on male reproductive toxicity in mice, and the results indicated that probiotic intervention improved PS-MP-induced reproductive toxicity by alleviating the inflammatory responses [53]. However, these beneficial effects must be weighed against emerging concerns. For example, probiotics that strongly bind MPs may alter their fate in the gut-MP interactions, thus potentially increasing the mucosal exposure, delaying transit, or affecting the release of bound toxicants. These risks remain hypothetical but highlight the need for a balanced, strain-specific assessment of probiotics.

### 5.3. Influence of dose, strain, and duration

The effectiveness of probiotics against microplastic toxicity widely varies by genus, and is further modulated by host-specific factors. For example, *Lactobacillus* strains are primarily known for their ability to produce extracellular polysaccharides (EPS) and promote gut barrier integrity, while *Bifidobacterium* species demonstrate potent immunomodulatory and antioxidant functions. Due to their spore-forming capacity, *Bacillus* strains often show resilience in hostile environments but may also exacerbate oxidative responses under MP exposure. Host-related factors such as age, baseline microbiota composition, diet, and immune status play a critical role in shaping the probiotic outcomes. These elements influence colonization, strain persistence, and immune response modulation, which are crucial to interpret variability across individuals and optimizing interventions in high-risk populations.

The ability of probiotics to mitigate MP toxicity depends on several factors, including the specific strain, administered dose, and duration of exposure. Evidence suggests that these protective effects are both dose-dependent and strain-specific. For instance, *Lactobacillus plantarum* has been shown to alleviate polystyrene MP-induced toxicity via multiple mechanisms, thus highlighting its potential role as a dietary approach to reduce the adverse impacts of MPs [44].

These findings highlight the need to select suitable probiotic strains and optimize the administration to counter MP-induced oxidative stress and inflammation. Chronic MP exposure may cause lasting microbiota changes, thus requiring prolonged probiotic use, especially in immunocompromised or microbiota-deficient hosts with a reduced microbial resilience. Understanding how probiotics persist and function in a disrupted and inflamed gut environment remains a critical knowledge gap.

Importantly, host-specific factors such as the baseline microbiota composition, immune status, and genetic background can strongly influence the efficacy of probiotic interventions. For instance, individuals with dysbiosis, immunosuppression, or gut barrier dysfunction may respond differently to the same probiotic regimen. These interindividual differences must be taken into account when designing future studies and interpreting the outcomes, particularly in vulnerable or chronically exposed populations.

## 6. Mechanistic insights into microplastic–probiotic interactions

### 6.1. How microplastics perturb probiotic and host physiology

#### 6.1.1. Disruption of microbial membranes and metabolism

MPs can adhere to bacterial cell surfaces via electrostatic and hydrophobic interactions, thus potentially altering the membrane permeability and inducing membrane stress [54]. These interactions are especially relevant for smaller MPs and NPs, which have a higher surface-area-to-volume ratio and an increased reactivity.

Upon contact with bacterial cells, MPs can disrupt membrane integrity and function through several physicochemical mechanisms. MPs may induce membrane depolarization and increase membrane permeability, thus leading to cytoplasmic leakage and compromised cell homeostasis [55]. Furthermore, MPs have been shown to alter nutrient uptake pathways and intracellular signaling processes, potentially by obstructing membrane transport systems or modifying surface charge interactions that are critical for the receptor activity [45].

#### 6.1.2. Induction of oxidative stress

MP exposure increases ROS production in probiotic bacteria. *In vitro* studies have demonstrated an elevated expression of antioxidant defense enzymes such as catalase, superoxide dismutase (SOD), and peroxidase in bacteria grown in the presence of MPs [56]. Persistent oxidative stress can lead to DNA damage, protein denaturation, and lipid peroxidation, thus compromising the bacterial fitness and function.

#### 6.1.3. Host-mediated dysregulation

Recent evidence from Wu *et al*. (2025) demonstrated that UV-aged MPs, particularly polyolefins such as polyethylene and polypropylene, undergo significant surface chemical changes including oxidation and fragmentation [57]. These aged MPs exhibit a greater interaction potential with gut microbiota and digestive components compared to virgin MPs. This highlights the importance of considering environmentally aged MPs in toxicological assessments and probiotic interaction studies.

*In vivo*, MPs compromise the gut epithelial integrity and stimulate local inflammation, thereby indirectly creating a hostile environment for probiotic colonization [58]. MPs can activate inflammatory pathways (*e.g*., NF-κB, TLR4 signaling) and reduce mucin production [51],[59], thus weakening the niche that probiotics rely on for adhesion and activity. Furthermore, MPs often carry adsorbed environmental contaminants such as phthalates, bisphenol A, or heavy metals, thus adding a secondary toxic burden that may synergistically impair probiotic function [60].

### 6.2. Probiotic countermeasures against microplastic toxicity

**Figure 2.**
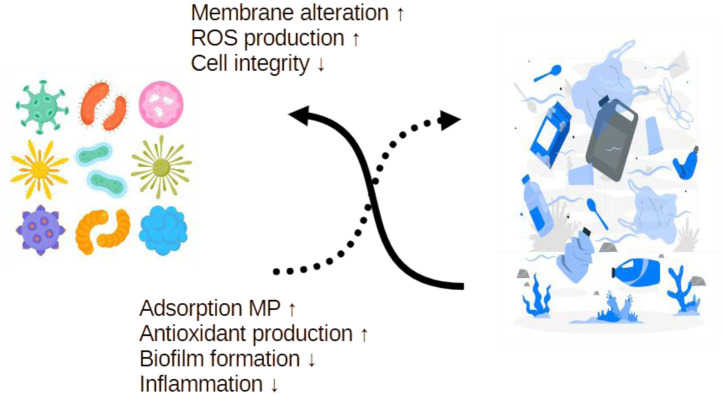
Bidirectional interactions between microplastics and microorganisms. MPs can disrupt microbial cell functions by inducing membrane alteration, increasing ROS production, and compromising cell integrity. In contrast, certain microorganisms, including probiotics, may exert modulatory effects on MPs through adsorption, antioxidant production, reduced biofilm formation, and attenuated inflammatory responses.

Despite the challenges posed by MPs, probiotics exhibit several adaptive and protective mechanisms that may mitigate MP-induced damage (Figure 2).

#### 6.2.1. Adsorption and aggregation of MPs

The mechanisms through which specific probiotics interact with different types of MPs are increasingly understood as being strain-, polymer-, and surface-dependent. For example, the capacity of *Lactobacillus* and *Bifidobacterium* strains to adsorb MPs is closely linked to their surface features such as teichoic acids, lipoteichoic acids, surface-layer proteins (SLPs), and extracellular polysaccharides (EPS). These components influence electrostatic interactions and the hydrophobic affinity, which are critical to bind MPs of various compositions. The binding efficacy may vary based on the MP polymer type (*e.g*., polystyrene vs polyethylene), particle size, and the extent of weathering, which alters the surface roughness and functional groups. Probiotics with high EPS production, such as *Lactiplantibacillus plantarum*, tend to show increased MP adsorption and aggregation. By contrast, strains which lack substantial surface adhesion molecules may be less effective [42],[61]. Furthermore, MPs with aged or oxidized surfaces interact more readily with bacterial EPS, thus suggesting that the physicochemical state of MPs is a crucial determinant of probiotic-MP interaction. These findings highlight the need for the targeted selection of probiotic strains based on the surface binding profiles and environmental MP characteristics.

While probiotics have shown a significant potential in mitigating MP-induced toxicity, it is crucial to acknowledge the 'dual-edged sword' nature of such interventions. Some probiotic strains may inadvertently facilitate the intestinal uptake of MPs or their adsorbed toxins. For instance, probiotics could prolong MP retention in specific gut regions through aggregation or enhanced mucosal adhesion, thus increasing the localized exposure. Additionally, by binding MPs, they may modify the bioavailability and release of co-contaminants such as persistent organic pollutants (POPs), antibiotics, or heavy metals [62]. These unintended consequences are especially relevant in hosts with an impaired gut barrier function or dysbiosis. Therefore, probiotic selection for MP mitigation should not solely focus on the adsorption capacity but also consider the risks of toxin mobilization and strain-specific immunological effects. A balanced evaluation of both protective and adverse impacts is essential to avoid overgeneralization and ensure a safe and effective clinical application.

Some probiotic strains, especially those which produce massive quantities of extracellular polymeric substances (EPS), can adsorb and aggregate MPs. EPS contains charged polysaccharides and proteins that interact with the plastic surface, thus forming microbe–MP complexes [42]. These aggregates may reduce free MPs in the gut lumen, promote fecal excretion, and limit epithelial contact. However, this aggregation could also enhance the MP bioavailability, especially for smaller particles or in conditions where the mucosal contact is prolonged. The potential for probiotics to inadvertently concentrate MPs in specific gut regions or to alter their distribution and retention remains underexplored and warrants further investigation [63].

#### 6.2.2. Biofilm-mediated protection

In the gut environment, an exposure to MPs can stimulate biofilm formation by certain probiotics [64]. These biofilms act as stress-adaptive structures, which provide physical protection against oxidative stress and enable an enhanced colonization of the intestinal epithelium [46]. Within these host-associated biofilms, cells coordinate through quorum sensing and share antioxidant enzymes and nutrients, thus promoting microbial survival and host barrier function under MP exposure. This differs from biofilms in environmental contexts, which primarily serve as platforms for plastic colonization or degradation [65].

#### 6.2.3. Modulation of host gut barrier and inflammation

Probiotics are known to support intestinal health by enhancing the barrier function and modulating the immune responses. They can upregulate the expression of tight junction proteins such as occludin, claudin-1, and Zonula Occludens-1, thereby helping to restore the epithelial integrity that may be disrupted by MPs [66]. In parallel, probiotics have been shown to suppress pro-inflammatory cytokines such as interleukin-6 (IL-6) and tumor necrosis factor-alpha (TNF-α), while promoting anti-inflammatory cytokines such as interleukin-10 (IL-10), thus contributing to a more balanced immune response [67]. Additionally, certain probiotic strains influence the balance between regulatory T cells (Treg) and T helper 17 (Th17) cells, thus promoting gut immune homeostasis and reducing inflammation-driven damage [68]. These multifaceted actions make probiotics a promising tool to counteract the immunotoxic and barrier-disrupting effects associated with MP exposure [69]. These actions may help reduce the gut permeability, limit the systemic translocation of MPs or endotoxins, and reestablish a microbiota-friendly environment.

#### 6.2.4. Antioxidant and metabolic contributions

Probiotic strains support antioxidant defenses by secreting compounds such as glutathione and exopolysaccharides that scavenge ROS, and by activating the Nrf2 pathway, which boosts enzymes such as SOD and Heme Oxygenase-1 [70]. *In vivo* studies in mice exposed to oxidative stressors, including MPs, linked these effects to lower MDA levels, reduced ROS in colon tissue, and an improved mitochondrial integrity [32],[70]. Table 1 summarizes the key probiotic strains evaluated for MP mitigation, along with their mechanisms of action and the experimental models used to assess their effects (Table 1).

**Table 1.** Probiotic strains, mechanisms of microplastic mitigation, and experimental models.

Probiotic Strain	Mechanism of MP Mitigation	Experimental Model	Ref
*Lacticaseibacillus paracasei* DT66	Adsorption and enhanced fecal excretion of PS-MPs; biofilm formation	*In vitro* (adsorption) and *in vivo* (mouse model)	[42]
*Lactiplantibacillus plantarum* DT88	Adsorption, inflammation reduction, restoration of barrier function	*In vitro* and *in vivo* (mouse model)	[42]
*Bacillus subtilis*	Increased biofilm formation and resilience under MP exposure	*In vitro*	[46]
*Bifidobacterium longum*	Immunomodulation, gut barrier reinforcement, antioxidant activity	*In vivo* (mouse model)	[44]
*Bacillus tropicus* ACS1	Altered growth kinetics, EPS production, antioxidant enzyme response	*In vitro* (fish gut-derived isolate)	[45]
*Bacillus cereus* SHBF2	MP degradation via bioflocculation and surface colonization	Environmental (aquaculture biofloc system)	[81]

Overview of selected probiotic strains investigated for their ability to mitigate MP toxicity. The table summarizes the key mechanisms involved—such as MP adsorption, antioxidant activity, or immune modulation—and the experimental models (*in vitro*, *in vivo*, or environmental) used to assess their effects. These strain-specific interactions highlight the potential of targeted probiotics for both health-oriented and environmental applications.

## 7. Probiotics as a mitigation strategy for microplastic toxicity (human health applications)

MPs have become an unavoidable component of the human exposome [71], entering the body primarily via ingestion and, to a lesser extent, inhalation.

### 7.1. Target populations and exposure contexts

While exposure to MPs is widespread across all populations, certain groups face disproportionately higher risks due to lifestyle, occupational, or geographical factors. High seafood consumers, particularly those living in coastal regions, are more likely to ingest MPs due to the documented contamination of fish and shellfish species [72]. Populations which heavily rely on packaged or processed foods, especially those stored in plastic containers or heated in plastic packaging, are also at a greater risk of dietary MP exposure [30]. Workers in plastic manufacturing, recycling, or waste treatment facilities may inhale or ingest MPs due to the constant proximity to airborne and surface-bound particles [73]. Infants and children represent another vulnerable group; studies have shown that infant formula prepared in polypropylene bottles can release millions of MP particles per liter, and toys or pacifiers made from plastic may add to the burden [74]. Finally, pregnant women are a population of growing concern, as recent evidence suggests that MPs can cross the placental barrier [75], thus raising potential risks for fetal exposure and developmental impacts [76].

### 7.2. Potential probiotic intervention strategies

Supplementation with specific probiotic strains offers a practical and scalable approach to mitigate MP-induced toxicity, particularly in populations with a high or chronic exposure. Targeted probiotic supplementation may effectively reduce MP toxicity. Strains such as *Lacticaseibacillus paracasei* DT66 and *Lactiplantibacillus plantarum* DT88 enhance MP excretion and alleviate inflammation [42]. Others, such as *Bacillus subtilis* and *Bifidobacterium longum*, support the gut barrier integrity and antioxidant defenses [44],[45],[69]. Effects are strain-specific and dose-dependent, thus emphasizing the need for evidence-based selection and formulation. Probiotics offer a promising, accessible strategy to protect the high-risk populations. Moreover, personalized approaches—such as matching probiotic strains to an individual's microbiota profile—may be essential to achieve consistent benefits. A “one-size-fits-all” approach is unlikely to be effective, given the high variability in microbiota composition, mucosal immunity, and intestinal transit time across individuals and populations. An integration of microbiome diagnostics could guide more targeted, responsive probiotic interventions in clinical or high-risk settings.

Combining probiotics with prebiotics (*e.g*., inulin, fructo-oligosaccharides, resistant starches) may enhance their colonization and functional impact. Some prebiotics may also bind MPs or modulate their gut transit time. Synbiotic strategies could offer enhanced resilience by supporting both beneficial microbes and gut integrity [77].

Future strategies to optimize probiotic interventions against MP toxicity may involve personalized and advanced approaches. These include selecting probiotic strains based on individual microbiome profiles to improve the host-specific compatibility and therapeutic efficacy. Moreover, tailored dosing regimens could be developed for individuals with chronic or occupational MP exposure, thus ensuring a consistent and targeted protection. Additionally, the use of multi-strain formulations or next-generation probiotics, such as *Akkermansia muciniphila* and *Faecalibacterium prausnitzii*, may offer superior anti-inflammatory effects and mucosal repair capacities, thus making them promising candidates for future clinical and preventive applications.

### 7.3. Challenges and opportunities for clinical translation

Despite promising results in preclinical models, translating probiotic interventions for MP exposure into clinical practice faces several hurdles. One major limitation is the lack of human trials, which are essential to validate the efficacy and safety. Future strategies may require personalized approaches, such as selecting probiotic strains based on individual microbiome profiles and tailoring dosing protocols for chronically exposed individuals, which include those in high-risk occupations.

Another challenge lies in the dosing and formulation. Probiotics effective in animal models may not yield the same results in humans due to factors such as gastric pH, intestinal transit time, and dietary influences. Advanced delivery systems, such as microencapsulation, could enhance the probiotic survival and colonization, thus improving the therapeutic outcomes.

Finally, regulatory and quality control issues remain significant barriers. In most countries, probiotics are marketed as foods or dietary supplements, with limited oversight on the strain specificity, viability, or functional claims. As their role in environmental health applications grows, stricter regulatory standards and robust clinical validation will be necessary to ensure consistency, safety, and efficacy.

In addition to delivery challenges, regulatory and commercial pathways for MP-targeted probiotics remain underdeveloped. Most current probiotic products are regulated as foods or supplements, with minimal oversight of strain identity, viability at time of use, or claimed function. For MP mitigation to become a credible indication, new regulatory frameworks must be established and should be supported by robust clinical evidence and risk–benefit analyses.

To bridge the gap toward clinical application, specific research priorities must be emphasized. First, validated biomarkers of MP exposure and probiotic efficacy are urgently needed. Potential candidates include fecal or urinary MP levels, markers of intestinal permeability (*e.g*., zonulin, LPS), systemic inflammation (*e.g*., CRP, IL-6), and oxidative stress (*e.g*., MDA, 8-OHdG). Second, pilot clinical trials should focus on high-risk populations, such as workers in plastic-intensive industries, individuals with high seafood consumption, or those with existing gut inflammation. These trials should apply stratified designs to account for microbiota composition, immune status, and the genetic background. Additionally, integrating omics technologies will provide insight into the mechanistic pathways affected by MPs and modulated by probiotics. Finally, randomized controlled trials using strain-specific formulations, combined with advanced delivery systems (*e.g*., encapsulation, colon-targeted release), are required to validate the safety, efficacy, and optimal dosing.

The following are among the proposed recommendations: (i) developing standardized product labeling for MP-targeted effects (*e.g*., “supports gut barrier under environmental stress”); (ii) encouraging pre-market registration or monographs of strains used in MP detoxification; (iii) testing encapsulated or acid-resistant delivery systems that ensure colon-specific release and viability under gastric conditions; and (iv) supporting public–private partnerships to fund early-phase clinical trials focused on MP-related endpoints.

## 8. Knowledge gaps and open questions–conclusion

Research on MPs and probiotics is still emerging but highlights a promising avenue for mitigating MP-induced health risks and supporting environmental applications. Preliminary *in vitro* and *in vivo* studies show that certain probiotics can adsorb MPs [42], enhance the gut barrier function [69], modulate oxidative stress [44], and promote the fecal elimination of MPs, thus suggesting protective roles against MP-driven dysbiosis and systemic inflammation. Nonetheless, several critical gaps must be addressed to translate these early findings into practical applications.

A primary challenge stems from the limitations of experimental models, which typically used pristine MPs with uniform properties, while real-world MPs are highly heterogeneous in size, shape, polymer composition, and surface chemistry, and are often altered by environmental exposure and the formation of eco-coronas [78]. This discrepancy questions the ecological and biological relevance of current data. Future research must incorporate environmentally sourced and aged MPs to better reflect the physicochemical diversity encountered in natural settings. Additionally, the exposure conditions represent a significant gap. The concentrations used in the experimental models often far exceeded the estimated human exposures, thus complicating the extrapolation to real-world scenarios. Chronic low-dose exposure studies, which are aligned with dietary MP intake, are underrepresented. Moreover, many *in vitro* systems lack critical physiological features such as mucus secretion, peristalsis, and host–microbe interactions, although advanced models such as gut-on-chip systems offer promising alternatives [79]. Standardizing the exposure categories (low, moderate, high) and developing harmonized protocols will be essential to enhance the reproducibility and comparability across studies.

In addition to the experimental design, host-specific factors play a crucial role in modulating the susceptibility to microplastic toxicity and the effectiveness of probiotic interventions. The individual responses are shaped by variables such as age, sex, health status, baseline microbiota, and genetic background, yet these elements are often overlooked in the current studies. To better account for this variability, future clinical and translational research should stratify the participants based on the microbiome composition, inflammatory markers, or immune profiles. Ultimately, personalized, microbiome-informed strategies may prove more effective than the one-size-fits-all approaches.

Critically, no human clinical trials have evaluated the impact of probiotic supplementation on MP-related health outcomes. Preclinical models provide valuable mechanistic insights but cannot substitute for human data. Translational research must prioritize the development of validated biomarkers of MP exposure and toxicity, such as fecal or urinary MP levels, gut barrier integrity, and systemic inflammation markers. Testing strain-specific probiotic formulations under realistic exposure conditions, combined with the stratification of participants based on host characteristics, will be key steps in building evidence-based recommendations. Moreover, observational studies which correlate the MP burden with gut health outcomes could lay important groundwork for future intervention trials.

While probiotics present promising protective mechanisms against MP-induced toxicity, their interactions with MPs also pose potential risks. Certain strains, such as *Lactiplantibacillus plantarum* DT88, effectively adsorb MPs and promote their fecal excretion [42], while *Bifidobacterium longum* supports the gut barrier function [69], and other strains mitigate oxidative stress and modulate immune responses [44]. However, these beneficial effects are counterbalanced by concerns that probiotics could act as vectors for harmful co-contaminants such as heavy metals or persistent organic pollutants [36],[63]. While protective in some contexts, biofilm formation might prolong MP retention within the mucosal environment, thus exacerbating the localized immune activation [68]. Moreover, strain-specific variability complicates this picture, with certain strains such as *Lactobacillus rhamnosus* GG demonstrating anti-inflammatory effects, while others, such as specific *Bacillus* strains, may aggravate oxidative stress under MP exposure. The environmental use of engineered probiotics, such as strains expressing PETase, raises concerns about potential ecological disturbances and the risk of horizontal gene transfer [80]. Navigating these trade-offs will require thorough toxicokinetic studies, careful strain selection based on selective MP binding capabilities, and strict regulatory oversight.

In conclusion, while the probiotic approach to mitigating MP-related toxicity holds considerable promise, its clinical translation depends on resolving key scientific, methodological, and regulatory challenges. Future studies must incorporate environmentally realistic MPs, standardized exposure models, and personalized intervention frameworks based on the host's microbiota profiles. Human clinical trials, supported by robust biomarker development and omics technologies, are urgently needed to move from proof-of-concept studies to evidence-based applications. Balancing the protective benefits of probiotics with their potential unintended effects will be critical to ensure that this promising strategy can be safely and effectively deployed in human health and environmental contexts.

## Use of AI tools declaration

The author declares that no Artificial Intelligence (AI) tools were used in the preparation of this article.
